# Antioxidant and hepatoprotective potential of *Lawsonia inermis* L. leaves against 2-acetylaminofluorene induced hepatic damage in male Wistar rats

**DOI:** 10.1186/s12906-017-1567-9

**Published:** 2017-01-18

**Authors:** Manish Kumar, Paramjeet Kaur, Madhu Chandel, Amrit Pal Singh, Arpana Jain, Satwinderjeet Kaur

**Affiliations:** 1 Department of Botanical and Environmental Sciences, Guru Nanak Dev University, Amritsar, 143005 Punjab India; 2 Department of Pharmaceutical Sciences, Guru Nanak Dev University, Amritsar, 143005 Punjab India; 3 Om Diagnostics, Amritsar, Punjab India

**Keywords:** *Lawsonia inermis*, 2-acetylaminofluorene, Lipid peroxidation, Hepatic damage, Hepatoprotective

## Abstract

**Background:**

*Lawsonia inermis* (Lythraceae) is an ethnomedicinal plant, traditionally known for curing several ailments such as skin diseases, bacterial infections, jaundice, renal lithiases and inflammation etc. The present work deals with assessment of in vitro antioxidant and in vivo hepatoprotective potential of butanolic fraction (But-LI) of *Lawsonia inermis* L. leaves.

**Methods:**

Antioxidant activity was evaluated using deoxyribose degradation, lipid peroxidation inhibition and ferric reducing antioxidant power (FRAP) assay. In vivo protective potential of But-LI was assessed at 3 doses [100, 200 & 400 mg/kg body weight (bw)] against 2-acetylaminofluorene (2-AAF) induced hepatic damage in male Wistar rats.

**Results:**

But-LI effectively scavenged hydroxyl radicals in deoxyribose degradation assay (IC_50_ 149.12 μg/ml). Fraction also inhibited lipid peroxidation and demonstrated appreciable reducing potential in FRAP assay. Treatment of animals with 2-AAF resulted in increased hepatic parameters such as SGOT (2.22 fold), SGPT (1.72 fold), ALP (5.68 fold) and lipid peroxidation (2.94 fold). Different concentration of But-LI demonstrated pronounced protective effects via decreasing levels of SGOT, SGPT, ALP and lipid peroxidation altered by 2-AAF treatment. But-LI administration also restored the normal liver architecture as evident from histopathological studies.

**Conclusions:**

The present experimental findings revealed that phytoconstituents of *Lawsonia inermis* L. possess potential to effectively protect rats from the 2-AAF induced hepatic damage in vivo possibly by inhibition of reactive oxygen species and lipid peroxidation.

## Background

Mutations resulting spontaneously or from environmental exposure may lead to cancer [1]. Chemical bonds in DNA molecule abide same laws likewise other chemicals existing at 37 °C in aqueous environment of cell. Likewise other molecules, existence of DNA also depends upon formation and breaking of bonds. So, it is not astonishing that DNA regularly endures various kinds of chemical damages due to spontaneous thermal effects and as result of attack of other reactive molecules [2]. Various physical and chemical agents (exogenous agents) causes damage to DNA, many of them are now documented as environmental carcinogens [2, 3]. Studies have shown that chemicals play an important role in the etiology of several kinds of human cancers [4, 5]. It is well-known that exposure to various hazardous chemicals occurs at very low doses, extends through longer time period of life and influence great part of population [6]. Tumor induction in workers exposed to coal tar in 1775 was the earliest known instance of environmental carcinogenesis documented. This very example of environmental carcinogenesis, later led to identification of various polycyclic hydrocarbons in coal tar. This also led to the finding of polycyclic hydrocarbons as skin carcinogens in laboratory animals. Other example was bladder carcinogenesis incidence among the workers working in the rubber and chemical industries. This led to the recognition of 2-naphthylamine as bladder carcinogen [2]. With advancement in the science, it is now well known that some of cancers are environmental in origin and can be related directly to different chemical exposures [7]. Humans are constantly exposed to plethora of xenobiotic chemicals and other related environmental pollutants which are hazardous to the health [8].

Liver is the main seat of xenobiotic metabolism and also carries out various functions in biotransformation including amino acid metabolism, lipid metabolism etc. [9–12]. Liver cancer is one of the most common malignancies occurring all over the world particularly in Asian and African countries [13]. Various risk factors linked with liver cancer are alcohol, food additives, aflatoxins, toxic chemicals from industries, pollutants etc. [14, 15]. More than 600 chemicals have been identified which can cause liver injury [16, 17]. 2-acetylaminofluorene (2-AAF) is one of the most studied chemical as model hepatocarcinogen. It was initially made as an insecticide, however its use was stopped because of its carcinogenic nature. It is an aromatic compound having solubility in organic solvents and remains insoluble in water [18–21]. 2-AAF induces its carcinogenic effects through metabolic activation *via* the mixed function oxidase system. Activation of 2-AAF leads to the formation of reactive electrophilic forms which react to form DNA adducts [22–24].

Nowadays, use of herbal medicines for curing variety of ailments is gaining popularity including liver diseases [25]. Number of reports are available in the literature which have shown hepatoprotective effects of natural plant products against various genotoxins, carcinogens and toxic substances including carbon tetrachloride (CCl_4_), paracetamol, 2-acetylaminofluorene (2-AAF), 7, 12-dimethylbenz(a)anthracene (DMBA), thioacetamide etc. [26–34]. *Lawsonia inermis* L. (*L. inermis*) commonly known as Henna or Mehandi belongs to family Lythraceae. Traditionally, the plant is known for its medicinal properties for the cure of renal lithiases, jaundice, to heal wounds, prevent skin inflammation etc. [35–38]. It is also used by some Nigerian tribes as a therapy against poliomyelitis and measles [39]. *L. inermis* was reported to contain various phytoconstituents such as chlorogenic acid, ferulic acid, isoferulic acid, gallic acid, o-coumaric acid, m-coumaric acid, myricetin, naringenin-7-o-rutinoside, quercetin, (+)-catechin, (−)-catechin gallate, (−)-epicatechin gallate, vitexin-2′-o-rhamnoside etc. [40]. Hsouna et al. [41] reported phytoconstituents *viz*. lawsoniaside, lalioside, luteolin-7- O-β -D-glucopyranoside, 2,4,6-trihydroxyacetophenone-2-O-β-D-glucopyranoside, 1,2,4-trihydroxynaphthalene-1- O-β-D-glucopyranoside from *L. inermis* leaves. *L. inermis* showed numerous medicinal properties *viz.* antimutagenic, anticlastogenic, analgesic, anti-inflammatory, antipyretic activities etc. [42–44]. Phytoconstituents from *L. inermis* leaves were reported to possess immunomodulatory activity [45]. Kaur et al. [46] carried out toxicity studies on ethanolic extract of *L. inermis* leaves using albino Wistar rats and reported that administration of rats with 80% ethanolic extract posed no toxicity in the tissues of the organs up to dose of 500 mg/kg bw. Another study by Alferah [47] reported that administration of *L. inermis* leaf solution (200 mg/kg/day) to the rats for 42 days did not induce any toxicity in liver, kidney and spleen tissue sections. Selvanayaki and Ananthi [48] reported hepatoprotective effects of aqueous extract of *L. inermis* against paracetamol induced hepatic damage in male Albino rats. Hossain et al. [49] studied hepatoprotective activity of *L. inermis* leaves against carbon tetrachloride induced liver damage in Wistar albino rats. Dasgupta et al. [50] reported anticarcinogenic activity of Henna leaves against benzo(a)pyrene induced forestomach as well as against 7,12 dimethylbenz(a)anthracene (DMBA)-initiated and croton oil-promoted skin papillomagenesis. In our previous reports [51, 52], we reported extract/fractions of *L. inermis* with antioxidant, antiproliferative and apoptosis inducing activity. Since But-LI fraction was found to exhibit high antioxidant activity and is rich in various polyphenolic phytoconstituents *viz*. gallic acid, catechin, chlorogenic acid, ellagic acid, kaempferol etc. [51], so we planned to investigate But-LI fraction from *Lawsonia inermis* L. for modulatory effects against the toxicity induced by 2-acetylaminofluorene (2-AAF) in male Wistar rats by assessing various serum and liver tissue parameters.

## Methods

### Chemicals

2,4,6-tripyridyl-*s*-triazine (TPTZ), Malondialdehyde (MDA), Deoxyribose, 2-acetylaminofluorene (2-AAF) were purchased from Sigma Chemical Co. (St Louis, MO, USA). Ascorbic acid and Sodium dodecyl sulfate (SDS) were purchased from Hi-Media, Mumbai, India. All other chemicals used in the present experimental study were of AR grade.

### Collection of plant material and preparation of But-LI fraction

The plant material was purchased from local market (Majeeth mandi, Amritsar), identified and authenticated by Dr. A. S. Soodan, Assoc. Prof., Department of Botanical and Environmental Sciences, Guru Nanak Dev University, Amritsar. The voucher specimen (no. 6773) has been kept in the Herbarium of the Department. Leaves were washed to ensure that they become free from any kind of dirt as well as other foreign particles and dried in shade. Leaves (3 kg) were grounded to fine powder and extracted at room temperature to obtain butanolic fraction (But-LI) as described in Kumar et al. [51].

### In vitro antioxidant assays

#### Deoxyribose degradation assay

Deoxyribose degradation assay was carried out by the method of Halliwell et al. [53] and Arouma et al*.* [54] with slight modifications. In this assay, EDTA (1 mM), FeCl_3_ (10 mM), hydrogen peroxide (10 mM), 2-deoxyribose (10 mM), test sample (1 ml), phosphate buffer and Ascorbic acid (1 mM) were mixed in the test tubes and the contents of reaction mixture were incubated at 37 °C for 1 h. After incubation, 1 ml of above mixture was taken and mixed with 1 ml of 2-thiobarbituric acid (TBA) and tricholoacetic acid (TCA) each. Finally reaction mixture was heated at 80 °C on water bath for 1.5 h. Final absorbance of the pink chromogen formed was taken spectrophotometrically at 532 nm using Elisa reader. Rutin was used as antioxidant standard.

Percent hydroxyl radical scavenging potential was calculated by formula as given below:

$$ \mathrm{Radical}\ \mathrm{scavenging}\ \mathrm{activity}\ \left(\%\right) = {\mathrm{A}}_0 - {\mathrm{A}}_1/{\mathrm{A}}_0 \times 100 $$

where,

A_0_ is the absorbance of reaction mixture + vehicle solvent,

A_1_ is the absorbance of reaction mixture + test sample.

### Lipid peroxidation inhibition assay

A modified thiobarbituric acid reactive species (TBARS) assay [55] was performed to determine the lipid peroxides produced using egg yolk homogenate as lipid rich media [56]. Various concentrations of test sample were added to the test tubes containing egg homogenate (0.5 ml of 10% v/v). About 50 μl of FeSO_4_ solution was added to the test tubes to induce lipid peroxidation and incubated test tubes at 37 °C for 30 min. After ½ hour, 20% acetic acid (pH 3.5), 0.8% of TBA in 1.1% SDS and TCA (20%) were added. All the contents of the tubes were mixed properly and heated at 95 °C for 1 h. After heating, test tubes were cooled, followed by addition of 5 ml of butanol and centrifuged at 1036 × *g* for 10 min. The absorbance was taken at 532 nm (Systronics 2202 UV–Vis Spectrophotometer). Trolox was used as antioxidant standard.

Inhibition of lipid peroxidation (%) was calculated using the formula as given below:

$$ \mathrm{Radical}\ \mathrm{scavenging}\ \mathrm{activity}\ \left(\%\right) = \left(1\ \hbox{-}\ \mathrm{E}/\mathrm{C}\right) \times 100 $$

where,

C is the absorbance of fully oxidized control,

E is the absorbance in the presence of test sample.

### Ferric reducing antioxidant power (FRAP) assay

FRAP assay was carried out by the method of Benzie and Strain [57]. FRAP reagent was prepared by mixing acetate buffer, TPTZ solution and FeCl_3_.6H_2_O solution in the ratio of 10:1:1. About 3 ml of FRAP reagent was dispensed into the test tubes followed by addition of 300 μl of test sample. Test tubes were shaken to mix the content well and incubated at 37 °C for 10 min. Absorbance of the reaction mixture was taken at 593 nm (Systronics 2202 UV–Vis Spectrophotometer, India). Trolox was used as standard antioxidant. Increase in the absorbance of the reaction mixture as compared to control is considered as increase in the reducing potential of test sample.

### In vivo hepatoprotective activity

#### Experimental animals

All the in vivo work was done as per the guidelines of Committee for the Purpose of Control and Supervision of Experiments on Animals (CPCSEA), Ministry of Environment and Forests, Government of India for housing and experimentation on animals and the study was approved by the Institutional Animal Ethics Committee of Guru Nanak Dev University, Amritsar (226/CPCSEA/2014/08). Male Wistar rats weighing 240–280 g were used in this study and were procured from the animal house facility of Indian Institute of Integrative medicine (IIIM), Jammu (India). After the procurement, rats were kept in the polypropylene cages provided with paddy husk bedding. The temperature was maintained at 25 ± 2 °C along with a 12 h light and 12 h dark cycle in the animal house of Guru Nanak Dev University, Amritsar (Punjab). All the animals were fed on standard pellet diet and water *ad libitum* and allowed to acclimatize for two weeks before the start of experimentation.

### Experimental design

**Fig. 1:**
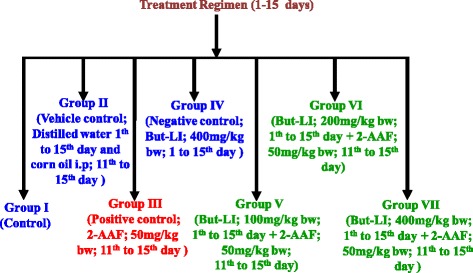
Diagrammatic representation of treatment schedule

2-acetylaminofluorene (2-AAF) induced hepatic damage model was used to evaluate hepatotoxicity [58]. The animals were randomly divided into seven groups (Fig. 1), each containing four rats (*n* = 4). The experimental protocol was of total 15 days time period. Group I served as control group put on standard pellet diet. Group II served as vehicle control and animals received distilled water via oral route (1st to 15th day) and corn oil injection intraperitoneally (i.p.) from 11th to 15th day. Group III served as positive control group was treated with 2-acetylaminofluorene (2-AAF) (50 mg/kg bw; i.p.) for 5 consecutive days (11th to 15th day). Group IV (Negative control) was treated with highest dose of plant extract alone (But-LI; 400 mg/kg bw) from day 1st to 15th, Group V to VII were given 3 doses of plant extract (100, 200 and 400 mg/kg bw from day 1st to 15th) and toxicant 2-AAF (50 mg/kg bw; intraperitoneally from day 11th to 15th).

### Preparation of liver homogenate

After completion of treatment period, all the rats were euthanized by cervical dislocation. Livers of the animals were perfused immediately in ice cold solution of 0.9% NaCl. Livers were then made free from other kind of tissues and rinsed in chilled buffer (0.15 M KCl + 10 mM Tris-HCl, pH 7.4). After that livers were weighed immediately and finally homogenized in ice-cold Tris-KCl buffer to yield 10% (w/v) homogenate using homogenizer.

### Biochemical analysis

#### Serum parameters

Blood samples were taken using retro-orbital puncture after anesthetizing the rats. Briefly, blood was allowed to stand for sometime followed by centrifugation at 2400 rpm for 20 min. Clear supernatant so obtained was designated as serum. Various serum parameters *viz.* Serum glutamate oxaloacetate transaminase (SGOT), Serum glutamate pyruvate transaminase (SGPT) and Serum alkaline phosphatase (ALP) were measured on Autoanalyzer (Erba Mannheim XL-640) using kits (Erba Mannheim XL System Packs).

### Determination of lipid peroxidation

Lipid peroxidation was determined in terms of the formation of thiobarbituric acid reactive species (TBARS) [59]. In order to measure TBARS, liver homogenate (0.5 ml) was added to the TBA reagent (20% TCA, 0.5% TBA, and 0.25 N HCl) in the test tubes. The contents of test tubes were mixed properly and heated at 80 °C for 30 min. After the incubation, test tubes were cooled to room temperature and final absorbance was taken at 532 and 600 nm. The amount of TBARS was expressed as MDA content (μ mol MDA eq/g of tissue) from the calibration curve obtained using malondialdehyde (MDA) as standard compound.

### Histopathological studies

For histopathological studies, liver tissues were fixed in 10% formalin solution. After that tissues were processed by routine histology method and finally embedded in paraffin wax. Tissue sections were then stained with haematoxylin and eosin stains. After the preparation of slides, the sections were studied for histopathological alterations under microscope equipped with camera. Coded histological samples of liver were scored for necroinflammatory score using the Ishak modified histological index grading [60].

### Statistical analysis

The results were expressed as the average and standard error/standard deviation. IC_50_ values were calculated using regression equation. The data was analyzed for statistical significance using analysis of variance (One-way ANOVA) and the difference among means was compared by HSD using Tukey’s test. The significance of results was checked at *p ≤ 0.05.

## Results

### In vitro antioxidant activity

**Fig. 2:**
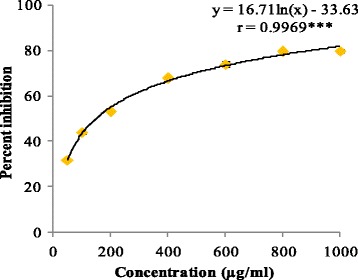
Hydroxyl radical scavenging activity of But-LI from *Lawsonia inermis* leaves in Deoxyribose degradation assay*.* ***represents significance at *p* ≤ 0.001

**Fig. 3:**
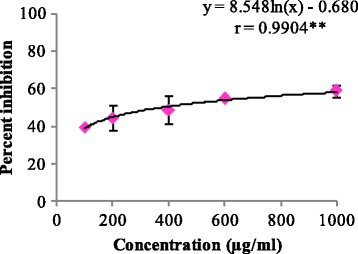
Lipid peroxidation inhibitory activity of But-LI from *Lawsonia inermis* leaves in Lipid peroxidation inhibition assay*.* **represents significance at *p* ≤ 0.01

**Fig. 4:**
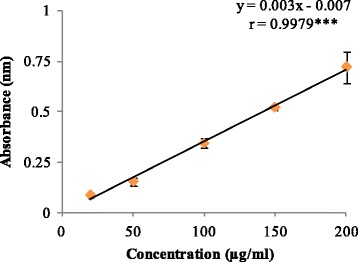
Reducing potential of But-LI from *Lawsonia inermis* leaves in FRAP assay*.* ***represents significance at *p* ≤ 0.001

In deoxyribose degradation assay, But-LI showed potent hydroxyl radical scavenging activity with percent inhibition of 31.81 at lowest tested concentration and 79.86% at highest tested concentration (Fig. 2). IC_50_ of 149.12 μg/ml was obtained from regression equation showed that fraction was more potent than standard rutin (IC_50_ 203.56 μg/ml). In lipid peroxidation inhibition assay, it showed moderate inhibition of 58.90% at highest concentration and 39.33% at lowest concentration. But-LI showed higher IC_50_ (375.73 μg/ml) than that of standard trolox (IC_50_ 136.47 μg/ml) (Fig. 3). But-LI displayed an absorbance of 0.72 nm in FRAP assay at highest tested concentration (Fig. 4). Standard compound trolox showed an increase in an absorbance of reaction mixture with 2.95 nm at same concentration (200 μg/ml). Results were dose-dependent as there was increase in absorbance with increase in the concentration of But-LI.

### In vivo hepatoprotective activity

#### Serum parameters

There was significant increase in the various serum toxicity markers *viz*. SGOT, SGPT and ALP on treatment with 2-AAF (Table 1). However, the vehicle control group and negative control group (But-LI treated) showed values which were not statistical different to the normal control group at *p* ≤ 0.05. The three different concentrations *viz*. 100 mg/kg, 200 mg/kg and 400 mg/kg bw of But-LI exhibited decrease in the values of these toxicity markers as compared to the toxicant i.e. 2-AAF treated group (Table 1).

**Table 1:** Effect of various treatments on hepatic parameters of male Wistar rats

S. No.	Group	SGOT (U/L)	SGPT (U/L)	ALP (U/L)
1.	Group I (Control)	^a^114.00 ± 11.284	^a^41.25 ± 7.632	^a^45.00 ± 8.286
2.	Group II (Vehicle Control)	^a^138.25 ± 6.652	^a^41.00 ± 1.414	^a^49.75 ± 12.038
3.	Group III [(Positive control; 2-AAF (50 mg/kg body weight)]	^b^253.25 ± 30.912	^b^71.00 ± 10.801	^b^256.00 ± 44.631
4.	Group IV (But-LI, 400 mg/kg body weight)	^a^121.50 ± 15.154	^a^40.50 ± 6.855	^a^44.75 ± 8.616
5.	Group V (2-AAF+ But-LI, 100 mg/kg body weight)	^a^138.50 ± 10.472	^a^43.75 ± 5.909	^a^46.25 ± 9.464
6.	Group VI (2-AAF+ But-LI, 200 mg/kg body weight)	^a^129.25 ± 10.242	^a^49.00 ± 9.931	^a^38.75 ± 4.500
7.	Group VII (2-AAF+ But-LI, 400 mg/kg body weight)	^a^118.50 ± 11.618	^a^38.00 ± 10.424	^a^31.00 ± 6.055
	F-ratio (6, 21)	39.02*	7.80*	75.51*
	HSD	35.91	18.76	42.82

Means within the column followed by different superscripts letters are significantly different at *p ≤ 0.05. Values expressed as Mean ± SD

### Determination of lipid peroxidation

Normal control group (I), vehicle control group (II) and negative control group (IV) showed TBARS value of 17.69, 15.87 and 14.15 μ mol MDA eq/g of tissue (Table 2). On the other hand, 2-AAF treated group (III) showed significant rise in the content of TBARS value of 52.07 μ mol MDA eq/g of tissue. 2-AAF and But-LI co-treated groups (V, VI & VII) showed dose dependent decrease in the TBARS values in comparison to 2-AAF treated group with value of 12.50 μ mol MDA eq/g of tissue at the highest concentration of 400 mg/kg bw (group VII) (Table 2).

**Table 2:** Effect of various treatments on lipid peroxidation status of male Wistar rats

S. No.	Group	Lipid peroxidation
		(μ mol MDA eq/g of tissue)
1.	Group I (Control)	^a^17.69 ± 3.460
2.	Group II (Vehicle Control)	^a^15.87 ± 3.591
3.	Group III [(Positive control; 2-AAF (50 mg/kg body weight)]	^b^52.07 ± 15.446
4.	Group IV (But-LI, 400 mg/kg body weight)	^a^14.15 ± 5.023
5.	Group V (2-AAF+ But-LI, 100 mg/kg body weight)	^a^22.70 ± 7.347
6.	Group VI (2-AAF+ But-LI, 200 mg/kg body weight)	^a^16.33 ± 3.967
7.	Group VII (2-AAF+ But-LI, 400 mg/kg body weight)	^a^12.50 ± 1.830
	F-ratio (6, 21)	14.753*
	HSD	16.534

Means within the column followed by different superscripts letters are significantly different at *p ≤ 0.05. Values expressed as Mean ± SD

### Histopathological studies

**Fig. 5:**
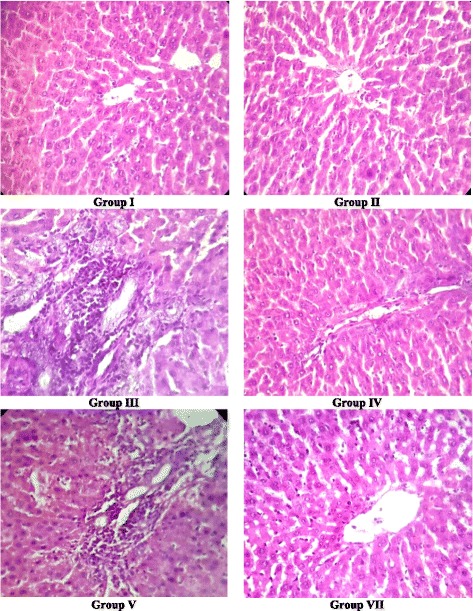
Histopathological examination of liver sections of rats belonging to different groups. *Group I* (Control); *Group II* (Vehicle control); *Group III* [(Positive control; 2-AAF (50 mg/kg bw)]; *Group IV* (But-LI, 400 mg/kg bw); *Group V* (2-AAF+ But-LI, 100 mg/kg bw); *Group VII* (2-AAF+ But-LI, 400 mg/kg bw)

Results showed that there was no damage to the liver in the normal control group (I), vehicle control group (II) and negative control group (IV) as the necroinflammatory score of these groups was zero. On the hand, histopathology examination of group III administered with 2-acetylaminofluorene (2-AAF) showed severe damage in the liver tissue with necroinflammatory score of 6/18. Group V, VI and VII administered with 100, 200 and 400 mg/kg bw of But-LI from *L. inermis* along with 2-AAF showed noticeable protection against 2-AAF induced liver damage. The necroinflammatory score was found to be 1 at the highest tested concentration of But-LI (VII; 400 mg/kg bw) pointing towards its protective effects (Table 3 & Fig. 5). Histopathological results revealed the protective potential of But-LI and support the results obtained from the serum and lipid peroxidation parameters.

**Table 3:** Modified hepatic activity index (HAI) grading (necroinflammatory scores)

S. No.	Group	Periportal or interface hepatitis (Piecemeal necrosis)	Confluent necrosis	Focal (spotty) lytic necrosis, apoptotic and focal inflammation	Portal inflammat-ion	Total score
1.	Group I	0	0	0	0	0/18
2.	Group II	0	0	0	0	0/18
3.	Group III	2	1	1	2	6/18
4.	Group IV	0	0	0	0	0/18
5.	Group V	1	0	1	1	3/18
6.	Group VI	0	0	0	1	1/18
7.	Group VII	1	0	0	0	1/18

## Discussion

Plant kingdom is regarded as gold repository of natural antioxidant constituents that can delay or prevent oxidation of other substances when ingested in daily diet [61]. During the last decade, bioprospection of phytoconstituents with nutritional and pharmaceutical importance is gaining popularity [62]. Numerous literature reports have demonstrated that medicinal plant extracts possess antioxidant and genoprotective activity [63–67]. Crude extracts or isolated molecules from medicinal plants not only possess antioxidant potential but they are even more potent than BHT, Vitamin E etc. in various in vitro experiments [68–70]. Mitochondrion is chief source of reactive oxygen species like superoxide anions (O_2_^.-^). Dismutation of superoxide anions radicals by superoxide dismutase enzyme leads to the formation of hydrogen peroxide (H_2_O_2_). Hydrogen peroxide undergoes interaction with transition metal ions *viz*. Fe^2+^ or Cu^+^ to generate hydroxyl radicals (OH^.^). Hydroxyl radicals (OH^.^) cause number of harmful activities such as initiating lipid peroxidation and causing alterations in DNA [71]. But-LI showed statistically significant dose-dependent hydroxyl radical scavenging potential of 79.86% at highest tested concentration. Analysis of one way ANOVA showed F-ratio of 50.29 which was found to be statistically significant at *p* ≤ 0.05. Regression analysis showed regression equation of y = 16.71ln(x)-33.63; *r* = 0.9969). The value of correlation coefficient was found to be significant at *p* ≤ 0.001. Since the fraction contained numerous polyphenolic constituents in appreciable amount as reported in our earlier publication [51], the hydroxyl radical scavenging activity of the fraction might be attributed to these compounds. Similar results were reported by Thind et al. [72] who investigated root extracts of *Schleichera oleosa* for antioxidant activity using deoxyribose degradation assay and reported extracts as good hydroxyl radical scavengers. Kaur and Arora [73] evaluated antiradical potential of methanol extract of *Chukrasia tabularis* leaves using deoxyribose degradation assay and reported that extract possessed promising hydroxyl radical scavenging activity in deoxyribose degradation assay.

Reactive oxygen species are mainly generated in the mitochondria as byproduct of cellular metabolic processes and can affect biomolecules by causing damage [74]. Reactive intermediates resulting from oxidative stress can target membrane bilayers by causing lipid peroxidation. Polyunsaturated fatty acids in the membranes undergo lipid peroxidation resulting in lipoperoxyl radical (LOO^.^) generation, which attack lipids to form lipid hydroperoxides (LOOH)^.^ and lipid radicals. Lipid hydroperoxides are known to be unstable and can give rise to peroxyl and alkoxyl radicals and decompose to produce various secondary products. The breakdown products of lipid peroxides include malondialdehyde, hexanal, 4-hydroxynonenal etc. which are highly reactive [75–78]. 4-hydroxynonenal is of electrophilic nature and reacts with glutathione, proteins and also with DNA at higher concentration [79, 80]. In the present investigation, it was found that But-LI moderately inhibited the lipid peroxidation dose dependently (y = 8.548ln(x)-0.680; *r* = 0.9904). The value of correlation coefficient was found to be significant at *p* ≤ 0.01. Earlier, we have reported that But-LI fraction harbours high amount of catechin, chlorogenic acid, ellagic acid and kaempferol while phytoconstituents such as gallic acid, epicatechin and quercetin were found to be present in moderate amount [51]. The lipid peroxidation inhibitory activity of the fraction might be due to various polyphenolic constituents present in it. Nakchat et al. [81] studied antioxidant activity including anti-lipid peroxidation activity of boiling water Tamarind seed coat extract and reported that extract effectively inhibited the lipid peroxidation. HPLC analysis of Tamarind seed coat extract showed presence of phenolics constitiuents such as (+)-catechin, (−)-epicatechin and procyanidin B2 which may be resposnsible for its antioxidant activities. Mulla and Swamy [82] studied antioxidant activity of polyphenolic extract of *Portulaca quadrifida* and reported that extract showed antilipid peroxidation activity of 71% with IC_50_ value of 370.33 ± 2.91 μg/ml. Results of FRAP assay revealed that But-LI fraction also possessed dose-dependent reducing potential. Analysis of results using one way ANOVA showed F-ratio of 49.94 which was found to be statistically significant at *p* ≤ 0.05. Regression analysis showed regression equation of y = 0.003×-0.007; *r* = 0.9979. The value of correlation coefficient was found to be significant at *p* ≤ 0.001. Reducing ability of the fraction may be due to polyphenols present in it. Singh et al. [83] studied leaf, fruit and seed extract of *Moringa oleifera* for antioxidant activity and reported that leaf extract possessed good reducing potential in reducing power assay. HPLC analysis of the extract demonstrated the presence of phenolic constituents such as gallic acid, chlorogenic acid, kaempferol, quercetin, ellagic acid, ferulic acid, and vanillin. Soobrattee et al. [84] studied various polphenolic phytochemicals for reducing potential in FRAP assay. Gallic acid, ellagic acid, chlorogenic acid, quercetin, kaempferol, (−)-epicatechin and (+)-catechin exhibited FRAP value of 5.25, 4.39, 3.22, 7.39, 1.95, 2.90 and 2.47 mmol Fe (II)/L respectively.

Several plant extracts and phytochemicals are reported to modulate the mammalian antioxidant enzymes system and provide protective effects against cellular damage [85–88]. Liver is the main organ responsible for detoxification processes occurring in the body. In the liver cells, endoplasmic reticulum is the primary site of metabolism. Hence, this metabolism is termed as hepatic metabolism. Besides liver, there are extrahepatic sites of metabolism which include organs such as lungs, kidney, skin, epithelial cells of gastrointestinal tract, adrenals and placenta [89–92]. Liver injury in response to various chemicals results in the leakage of serum enzymes into the blood circulation, thus causing increase in their level in the serum [93]. Sehrawat et al. [94] reported that 2-AAF administration to rats increased the level of SGOT and SGPT enzymes in serum. In another study, Hasan and Sultana [34] reported that 2-AAF treated rats demonstrated high level of serum aspartate aminotransferase (AST) and alanine aminotransferase (ALT). In the present investigation results obtained from serum toxicity markers such as SGOT, SGPT, ALP demonstrated significant increase on treatment with 2-AAF as compared to normal control. 2-AAF induced 2.22, 1.72 and 5.68 fold enhancements in SGOT, SGPT and ALP levels respectively. On co-administration of rats with 2-AAF and varying doses of But-LI, there was significant decrease in these serum parameters and the serum enzymes levels were restored towards normal control levels. The But-LI fraction alone did not induce any increase in the values of these markers and results were statistically not different to the normal control and vehicle control group at *p* ≤ 0.05, reflecting non-toxic nature of But-LI fraction.

A study carried out by Selvanayaki and Ananthi [48] reported aqueous extract of *Lawsonia inermis* seeds with potent hepatoprotective effects against paracetamol induced hepatic damage in male rats. Extract significantly reduced the levels of various serum enzymes viz. aspartate aminotransferase (AST), acid phosphatase (ACP), alkaline aminotransferase (ALT), alkaline phosphatase (ALP) etc. altered by paracetamol treatment. Recently, Mohamed et al. [95] reported hepatoprotective potential of methanol extract of *L. inermis* leaves against carbon tetrachloride (CCl_4_)-induced hepatic damage. It was found that extract treatment significantly protected rats from hepatic damage induced by CCl_4_.

Lipid peroxidation is critical marker of oxidative stress and is coupled with various diseases including cancer [96, 97]. Malondialdehyde (MDA) and lipid hydroperoxides are produced as the result of lipid peroxidation of polyunsaturated fatty acids [86, 92]. Results of the present investigation demonstrated that 2-AAF treatment resulted in 2.94 fold increase in the MDA level in rats. Further, treatment of rats with But-LI along with 2-AAF reversed the effect of 2-AAF as reflected from lower level of MDA. Our results are in agreement with previous studies [34, 88, 94, 95], in which natural plant products effectively reduced lipid peroxidation induced in response to various toxicants. Further, results of histopathological examination were also in concordance with results of other parameters and provided supportive evidence regarding protective potential of *Lawsonia inermis* (But-LI) fraction. It was found that 2-AAF administration to the male Wistar rats caused severe damage to the liver tissue, since it showed various histopathological alterations such as moderate piecemeal necrosis, mild confluent and spotty necrosis, moderate portal inflammation etc. with necroinflammatory score of 6 out of 18. The untreated, vehicle and negative control group rats did not demonstrate such pathologies in their liver tissue and necroinflammatory score was found to be zero. All the 3 doses (100, 200 and 400 mg/kg bw) provided protection against damage induced by 2-AAF with necroinflammatory score of 1 out of 18 at highest tested dose (400 mg/kg bw) and histoarchitecture of the animals in these groups was comparable to untreated control group. Hepatoprotection can be achieved either by reinstating the normal hepatic physiology or by diminishing the toxic damaging effect induced by toxicant [98]. The in vivo protective activity of But-LI of *Lawsonia inermis* against 2-AAF could be attributed to the various polyphenolic phytochemicals present in the fraction.

## Conclusions

The present experimental findings revealed that phytoconstituents of *Lawsonia inermis* L. possess potential to protect rats from the 2-AAF induced hepatic damage in vivo possibly by inhibition of radicals and lipid peroxidation.

## Abbreviations

2-AAF: 2-acetylaminofluorene
ALP: Alkaline phosphatase
CPCSEA: Committee for the purpose of control and supervision of experiments on animals
FRAP: Ferric reducing antioxidant power
MDA: Malondialdehyde
SGOT: Serum glutamic oxaloacetic transaminase
SGPT: Serum glutamic pyruvic transaminase
TBARS: Thiobarbituric acid reactive species
TPTZ: 2,4,6-tripyridyl-s-triazine
